# Modification of the eighth AJCC/UICC staging system for perihilar cholangiocarcinoma: An alternative pathological staging system from cholangiocarcinoma-prevalent Northeast Thailand

**DOI:** 10.3389/fmed.2022.893252

**Published:** 2022-09-30

**Authors:** Chaiwat Aphivatanasiri, Prakasit Sa-Ngiamwibool, Sakkarn Sangkhamanon, Piyapharom Intarawichian, Waritta Kunprom, Malinee Thanee, Piya Prajumwongs, Narong Khuntikeo, Attapol Titapun, Apiwat Jareanrat, Vasin Thanasukarn, Tharatip Srisuk, Vor Luvira, Kulyada Eurboonyanun, Julaluck Promsorn, Watcharin Loilome, Aileen Wee, Supinda Koonmee

**Affiliations:** ^1^Cholangiocarcinoma Screening and Care Program (CASCAP), Khon Kaen University, Khon Kaen, Thailand; ^2^Cholangiocarcinoma Research Institute, Khon Kaen University, Khon Kaen, Thailand; ^3^Department of Pathology, Faculty of Medicine, Khon Kaen University, Khon Kaen, Thailand; ^4^Department of Surgery, Faculty of Medicine, Khon Kaen University, Khon Kaen, Thailand; ^5^Department of Radiology, Faculty of Medicine, Khon Kaen University, Khon Kaen, Thailand; ^6^Department of Biochemistry, Faculty of Medicine, Khon Kaen University, Khon Kaen, Thailand; ^7^Department of Pathology, National University Hospital, Singapore, Singapore

**Keywords:** perihilar cholangiocarcinoma, eighth AJCC/UICC staging, KKU staging system, classification, growth pattern

## Abstract

**Aim:**

This study aims to improve the classification performance of the eighth American Joint Committee on Cancer (AJCC) staging system for perihilar cholangiocarcinoma (pCCA) by proposing the Khon Kaen University (KKU) staging system developed in cholangiocarcinoma-prevalent Northeast Thailand.

**Method:**

Four hundred eighty-eight patients with pCCA who underwent partial hepatectomy between 2002 and 2017 at the Srinagarind Hospital, Faculty of Medicine, Khon Kaen University, Thailand, were included. Overall survival (OS) related to clinicopathological features was analyzed using the Kaplan–Meier method. Logrank test was performed in univariate analysis to compare OS data of clinicopathological features to determine risk factors for poor survival. Significant features were further analyzed by multivariate analysis (Cox regression) to identify prognostic factors which were then employed to modify the eighth AJCC staging system.

**Results:**

Multivariate analysis showed that growth pattern (HR = 4.67–19.72, *p* < 0.001), moderately and poorly differentiated histological grades (HR = 2.31–4.99, *p* < 0.05 and 0.001, respectively), lymph node metastasis N1 and N2 (HR = 1.37 and 2.18, *p* < 0.05 and 0.01, respectively), and distant metastasis (HR = 2.11, *p* < 0.001) were independent factors when compared to their respective reference groups. There was a clear separation of patients with pCCA into KKU stage: I [OS = 116 months (mo.)], II (OS = 46 mo.), IIIA (OS = 24 mo.), IIIB (11 mo.), IVA (OS = 7 mo.), and IVB (OS = 6 mo.).

**Conclusion:**

The new staging system was based on the incorporation of growth patterns to modify the eighth AJCC staging system. The classification performance demonstrated that the KKU staging system was able to classify and distinctly separate patients with pCCA into those with good and poor outcomes. It was also able to improve the stratification performance and discriminative ability of different stages of pCCA classification better than the eighth AJCC staging system. Hence, the KKU staging system is proposed as an alternative model to augment the accuracy of survival prognostication and treatment performance for patients with pCCA.

## Introduction

Cholangiocarcinoma (CCA) is a cancer of epithelial origin arising from different locations within the intra- and extrahepatic biliary tree. The incidence is remarkably high in Asian countries, especially in Thailand, which has the highest reported incidence in the world (1, 2). The major risk factor for Thai patients with CCA is evidently associated with the liver fluke, *Opisthorchis viverrini* (3–5). *O. viverrini* has been reported to enhance cholangiocarcinogenesis via several carcinogenic mechanisms (6, 7). In general, late presentations with locally advanced or metastatic disease contribute to high mortality and dismal response after surgery (8–10). CCA is classified into three types based on anatomical localization, namely, intrahepatic (iCCA), perihilar (pCCA), and distal (dCCA) CCA. This study focused on pCCA because it is the commonest type with distinct epidemiology and dismal outcomes after treatment (9, 11).

Perihilar CCA has a reported incidence rate of approximately 50–70% of all CCA (12, 13). By definition, pCCA is a tumor that is located in the extrahepatic biliary tree proximal to the origin of the cystic duct. Proximally, the tumor extends up to the secondary branches of the right and left hepatic ducts and invades the liver parenchyma (14). This results in the formation of three major types of growth patterns, comprising intraductal (ID), periductal infiltrating (PI), and mass-forming (MF) growth types, similar to iCCA (15–18). In pCCA, PI and MF growth patterns have been reported as risk factors for poor survival, while the ID pattern favors a good prognosis of patients with CCA in general (18). The survival time of patients with pCCA is poor, with overall survival of about 12 months. The survival time can be prolonged to 40 months by post-operative palliative treatment (11). Almost all prognostic studies focused on patients who have undergone resection. The dismal post-operative outcomes are attributed to tumor recurrence and advanced stage at presentation (10, 11). Present studies propose adjuvant therapy as an additional treatment option. Rizzo and Brandi (19) updated adjuvant therapy for CCA, especially in pCCA cases, based on the results of phase III studies in three clinical trials analyzing adjuvant systemic therapy in resected bile duct tumors, namely, the BILCAP (United Kingdom), the BCAT (Japan), and the PRODIGE-12/ACCORD-18 (France). Although these studies showed good outcomes, only the BILCAP trial demonstrated statistically significant outcomes and safety. Moreover, adjuvant therapy is still debatable and controversial in terms of safety and treatment efficiency issues in the CCA medical community. Another alternative treatment is immunotherapy for advanced CCA treatment suggested by Rizzo et al. (20). They reported that immune checkpoint inhibitors (ICI) can provide effective treatment in phases I–III of advanced CCA. ICI is deemed a new strategy to combat advanced CCA and there remains room for investigations to confirm performance and safety. Therefore, effective stratification of patients with pCCA is crucial for precise prognostic discrimination to improve treatment performance.

The American Joint Committee on Cancer (AJCC) and The Union for International Cancer Control (UICC) staging system are one of the most common strategies for the stratification of cancer staging. The eighth edition of the AJCC/UICC Staging Manual was recently updated (21). Several studies comparing stratification performances of the seventh and eighth editions have suggested that the latter has improved classification (22–25). However, there have been observations that the overall performance of eighth AJCC staging to classify pCCA is not notably better. In fact, there are increasing reviews that the eighth AJCC has shown only slight improvement leading to calls for further refinements to improve the prognostic discriminative ability by applying prognostic factors (26–32). There are several reports of modifications of the eighth AJCC staging system using prognostic factors from multivariate analysis, such as invasive tumor thickness (ITT) (26, 27), tumor size (28), bilirubin level (29), and serum CA19-9 (30, 31), and creation of a new staging system (Mayo Clinic Staging System) (32). These modifications provide improvement in prognostication when compared to conventional classification by the eighth AJCC staging system.

This study, henceforth, aims to modify the eighth AJCC staging system for pCCA with the creation of an alternative staging system called the Khon Kaen University (KKU) staging system and to compare the prognostic and classifying ability of the two systems. All patients with pCCA with partial hepatectomy were stratified using the eighth AJCC staging system, and possible features significantly related to overall survival (OS) were analyzed by multivariate analysis to identify prognostic factors for poor survival. These prognostic factors were then employed to modify the eighth AJCC staging system to develop the KKU model.

## Materials and methods

### Patients

**Figure 1:**
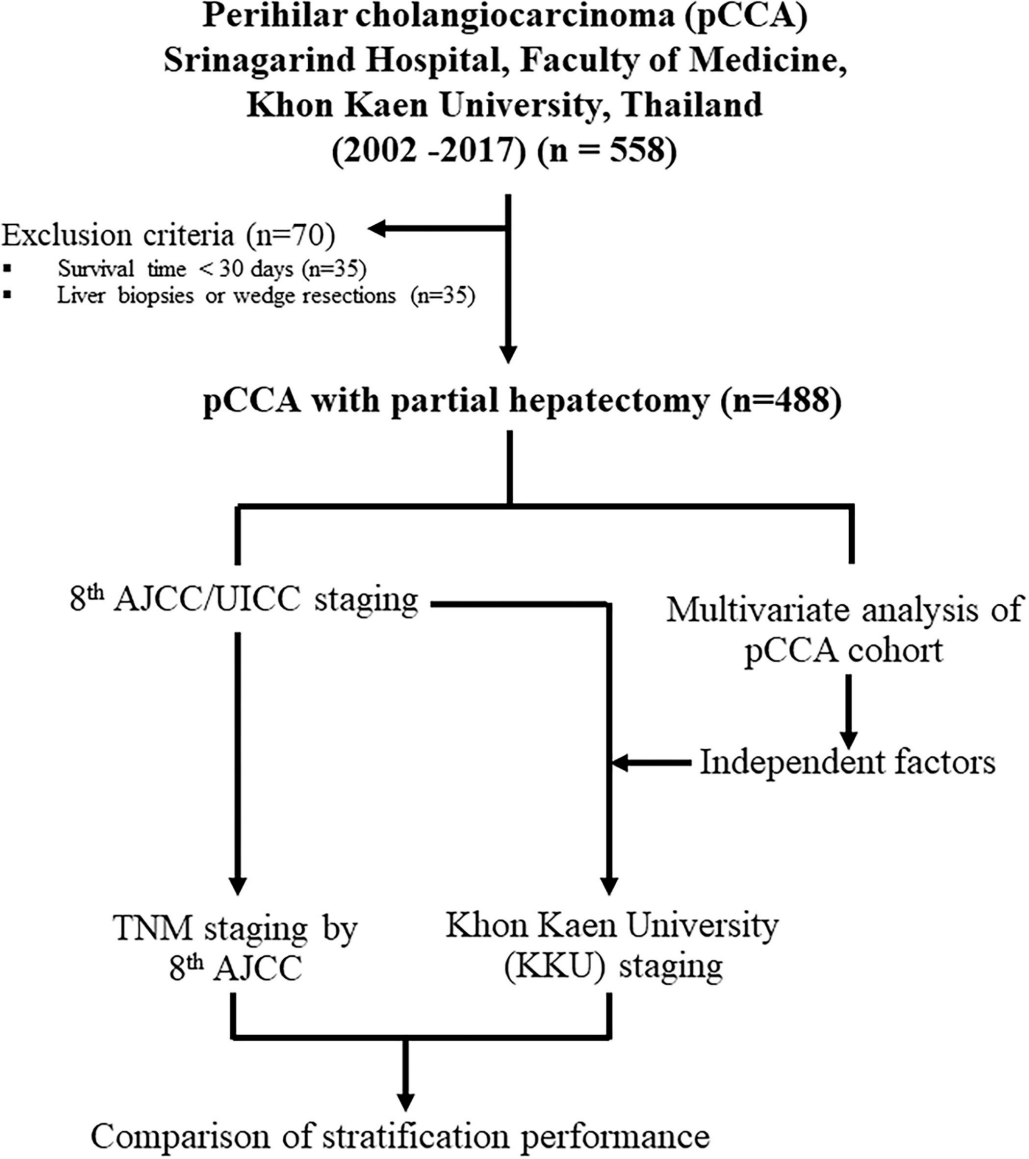
Schematic design for this study on the modification of the eighth edition AJCC/UICC staging system to the Khon Kaen University (KKU) staging system for the prognostic stratification and management of patients with perihilar cholangiocarcinoma in Northeast Thailand.

Between 2002 and 2017, 558 patients were diagnosed with pCCA at the Srinagarind Hospital, Faculty of Medicine, Khon Kaen University, Thailand. There were three groups of patients, namely, (i) those with liver biopsies or wedge resections (*n* = 35), (ii) those who survived less than 30 days after surgery with likely perioperative causes of death (*n* = 35), and (iii) those who underwent partial hepatectomies (*n* = 488). The first two groups were excluded. A final total of 488 patients was included. The experimental workflow is shown in Figure 1. This research was approved by the Ethics Committee for Human Research, Khon Kaen University (HE641499).

### Recorded data

Patients underwent hepatectomy with the *en-bloc* resection of the tumor and extrahepatic bile duct according to the Bismuth-Corlette classification. The extent of the tumor was evaluated with pre-operative imaging by computerized tomography (CT) and magnetic resonance cholangiopancreatography (MRCP) (33, 34). Lymph node (LN) sites, namely, the common hepatic artery (LN8), hepatoduodenal ligament (LN12), and posterior pancreaticoduodenal node (LN13), were regularly resected. Combined vascular resection and reconstruction to the remnant side of the liver were performed if the tumor involved the portal vein or hepatic artery. Intraoperative data collection comprised sex, age, hepatic resection region, sample diameter, tumor diameter, growth patterns (ID, PI, and MF), surgical margin, and characteristics of surrounding organs.

The liver specimens were examined with relevant tissue blocks taken by a pathologist for routine tissue processing. The tumor sampling consisted of at least one block per centimeter of the largest dimension of tumor. Formalin-fixed paraffin-embedded (FFPE) tissue blocks were sectioned at 5 microns and stained with hematoxylin and eosin (H&E). Pathological diagnosis was reviewed under the 2019 World Health Organization (WHO) classification criteria (35). Four pathologists conducted a double-blind microscopic review of the tissue slides, and a consensus diagnosis was taken. The following histomorphological data were recorded: growth patterns, histological type, histological grade, surgical margin, lymphovascular invasion, and lymph node metastasis. Distant metastasis status was retrieved from the medical records. Finally, the gross examination and pathological data were used to determine pathological staging following the eighth AJCC staging manual (21) (Table 1).

**Table 1:** Definition of TNM categories of the eighth AJCC/UICC staging manual.

**Tumor category (T)**	**Lymph node metastasis (N)**	**Distal metastasis (M)**
T*is* = Carcinoma in *situ*//high-grade dysplasia	N0 = Negative lymph node	M0 = No distant metastasis
T1 = Tumor confined to the bile duct, with extension up to the muscle layer or fibrous tissue	N1 = One to three positive lymph nodes	M1 = Distant metastasis
T2 = Tumor invades beyond the wall of the bile duct:	N2 = Four or more positive lymph nodes	
T2a = Tumor invades beyond the wall of the bile duct to surrounding adipose tissue		
T2b = Tumor invades adjacent hepatic parenchyma		
T3 = Tumor invades unilateral branches of the portal vein or hepatic artery		
T4 = Tumor invades the main portal vein or its branches bilaterally, or the common hepatic artery; or unilateral second-order biliary radicals with contralateral portal vein or hepatic artery involvement		

### Growth pattern proportion

The liver resection specimens were serially sectioned, photographed, and tumor growth pattern/s were recorded at the time of gross examination and subsequently confirmed histologically. The growth patterns were estimated as increments of 10% to establish the proportion of each pattern, namely, ID, PI, MF, or combination of patterns (ID+PI, ID+MF, PI+MF, and ID+PI+MF).

### Pathological diagnosis

There were four major histological types found in this study, namely, papillary adenocarcinoma, tubular adenocarcinoma, papillotubular adenocarcinoma, and adenocarcinoma, not otherwise specified (NOS). Papillary, tubular, and papillotubular adenocarcinomas were graded as well-or moderately differentiated carcinomas and diagnosed according to 2019 WHO classification criteria (35). Adenocarcinoma, NOS, was defined as poorly differentiated bile duct cancer lacking well-formed papillary or tubular formations.

### Statistical analysis

Only patients with complete datasets were included in the statistical analyses. Categorical data were reported as counts and percentages. Clinicopathological features were compared using chi-square tests (χ2-Test), and continuous variables were compared using Student's *t*-tests. Survival analysis using the Kaplan–Meier model was applied for OS calculation, and the Logrank test was used for the comparison of OS for each clinicopathological feature. Patients with perioperative causes of death were excluded from this analysis (survival time <30 days). For growth pattern estimation, 20% was used as a cut-off value as this figure showed significantly different OS between each pattern. Statistically significant features from the Logrank test were further examined by multivariate analysis. Cox regression model was performed to identify independent factors in multivariate analysis.

## Results

### Basic clinicopathological characteristics of patients with perihilar cholangiocarcinoma

A total of 488 patients underwent partial hepatectomy for pCCA. There were two types of pCCA comprising non-invasive (tumor *in situ, n* = 25) and invasive tumors (*n* = 463); the non-invasive category was used as the baseline for good survival of patients with pCCA. The clinicopathological features are shown in Table 2. The median age of 58 (range, 33–78) years was used to separate the patients into two groups of ≤ 58 years (*n* = 251, 51.4%) and >58 years (*n* = 237, 48.6%). There were 344 men (70.5%) and 144 women (29.5%).

**Table 2:** Clinicopathological features of patients with perihilar cholangiocarcinoma.

**Features**	***n* = 488**	**%**
**Age (year)**		
≤ 58	251	51.4
>58	237	48.6
**Sex**		
Male	344	70.5
Female	144	29.5
**Tumor size (range, 0.2–24 cm)**		
Tumor *in situ*	25	5.1
≤ 4	177	36.2
>4	155	31.8
Unknown	131	26.9
**Growth pattern**		
Tumor *in situ* (Intraductal growth, ID)	25	5.1
Intraductal growth (ID)	39	8
Periductal infiltrating (PI)	94	19.3
Mass forming (MF)	119	24.4
ID+PI	54	11.1
ID+MF	36	7.4
PI+MF	82	16.8
ID+PI+MF	39	8
**Surgical margin (R)**		
R0 (tumor *in situ*)	25	5.1
R0	180	36.9
R1	283	58
**Histological types**		
Tumor *in situ* (P or T)	25	5.1
Papillary (P)	196	40.2
Tubular (T)	180	36.9
Papillotubular (P+T)	19	3.9
Adenocarcinoma, NOS	68	13.9
**Histological grade**		
Well-differentiated (tumor *in situ*)	25	5.1
Well-differentiated	383	78.5
Moderately differentiated	56	11.5
Poorly differentiated	24	4.9
**T categories**		
T*is*	25	5.1
T1	40	8.2
T2a	125	25.6
T2b	197	40.4
T3	71	14.5
T4	30	6.2
**Lymph node metastasis (N)**		
N0 (tumor *in situ*)	25	5.1
N0	248	50.8
N1	182	37.3
N2	33	6.8
**Distant metastasis (M)**		
M0 (tumor *in situ*)	25	5.1
M0	415	85.1
M1	48	9.8

Gross examination revealed the median tumor size to be 4 cm (range, 0.2–24 cm). Based on tumor size, there were four groups, namely, ≤ 4 cm (*n* = 177, 36.2%), >4 cm (*n* = 155, 31.8%), tumor *in situ* (*n* = 25, 5.1%), and unknown size (*n* = 131, 26.9%). The tumors exhibited a spectrum of gross growth patterns ranging from tumor *in situ* with ID (*n* = 25, 5.1%) to ID (*n* = 39, 8%), PI (*n* = 94, 19.3%), MF (*n* = 119, 24.4%), and combinations of ID+PI (*n* = 54, 11.1%), ID+MF (*n* = 36, 7.4%), PI+MF (*n* = 82, 16.8%), and ID+PI+MF (*n* = 39, 8%). The surgical margin was assessed microscopically to be free from tumor, R0 (tumor *in situ, n* = 25, 5.1%), R0 (*n* = 180, 36.9%), and involved by tumor, R1 (*n* = 283, 58%).

Pathological examination revealed the following histological types: tumor *in situ* with either papillary or tubular adenocarcinoma (*n* = 25, 5.1%); papillary adenocarcinoma (P) (*n* = 196, 40.2%); tubular adenocarcinoma (T) (*n* = 180, 36.9%); papillotubular adenocarcinoma (P+T) (*n* = 19, 3.9%); and adenocarcinoma, NOS (*n* = 68, 13.9%). Histological grades comprised well- (tumor *in situ, n* = 25, 5.1%), well- (*n* = 383, 78.5%), moderately (*n* = 56, 11.5%), and poorly differentiated carcinomas (*n* = 24, 4.9%).

According to the eighth AJCC staging system (Table 1), T categories comprised T*is* (*n* = 25, 5.1%), T1 (*n* = 40, 8.2%), T2a (*n* = 125, 25.6%), T2b (*n* = 197, 40.4%), T3 (*n* = 71, 14.5%), and T4 (*n* = 30, 6.2%). Lymph node metastasis (N) following the eighth AJCC staging system was divided into four groups: N0 (tumor *in situ, n* = 25, 5.1%), N0 (*n* = 248, 50.8%), N1 (*n* = 182, 37.3%), and N2 (*n* = 33, 6.8%). Distant metastasis (M) comprised M0 (tumor *in situ, n* = 25, 5.1%), M0 (*n* = 415, 85.1%), and M1 (*n* = 48, 9.8%). The metastatic sites, which were confirmed non-local invasion, included gallbladder (*n* = 20), falciform ligament (*n* = 2), hepatoduodenal tissue (*n* = 5), omentum (*n* = 10), peritoneum (*n* = 5), diaphragm (*n* = 5), and skull (*n* = 1). All data were further analyzed for survival times (univariate analysis by Logrank test) and prognostic risk factors (multivariate analysis by Cox regression).

### Survival and univariate analysis of possible risk factors of patients with perihilar cholangiocarcinoma

**Figure 2:**
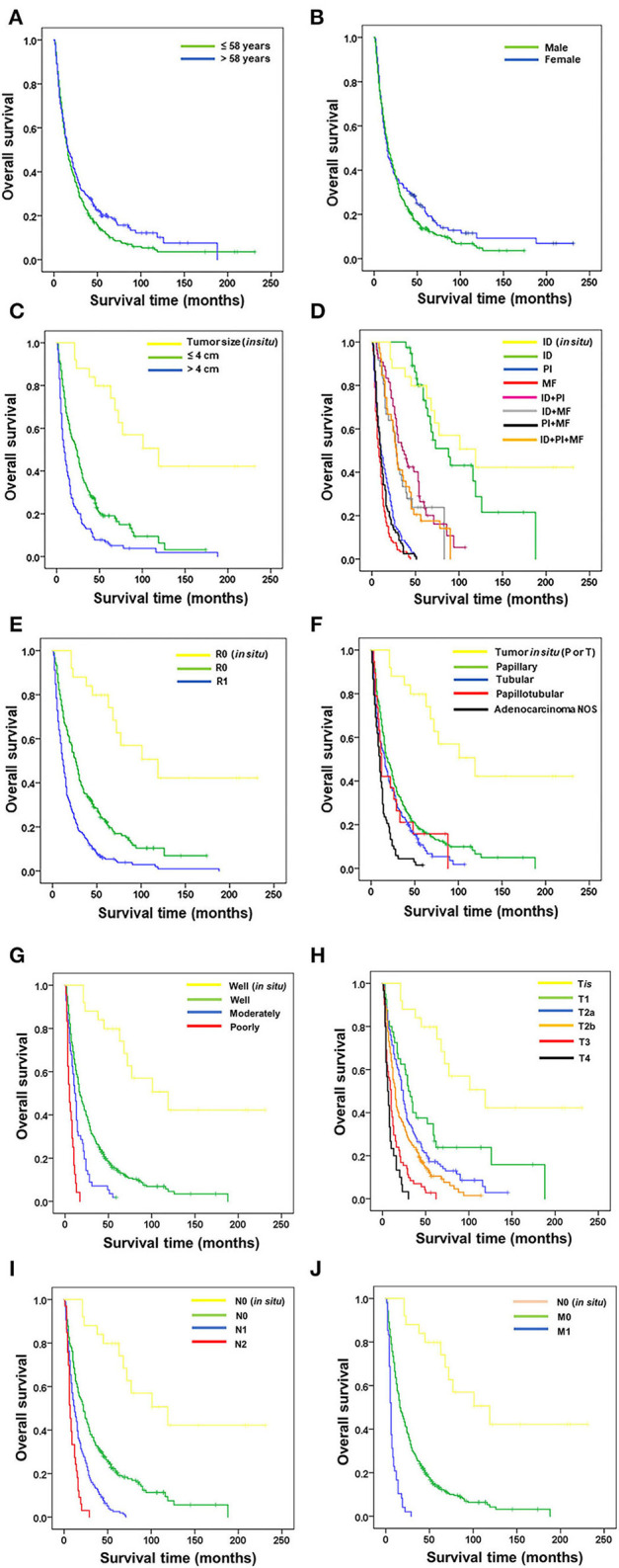
Correlation of overall survival of patients with perihilar cholangiocarcinoma with clinicopathological features. Correlation of OS with **(A)** age, **(B)** gender, **(C)** tumor size, **(D)** growth patterns, **(E)** surgical margin, **(F)** histological type, **(G)** histological grade, **(H)** T stage, **(I)** lymph node metastasis, and **(J)** distant metastasis.

The OS and 5-year survival rate (5y) of the 488 patients with pCCA were analyzed with the Kaplan–Meier model and the comparison of OS for the various clinicopathological features with the Logrank test. There was no significant difference in the survival times of age and gender of patients. Therefore, neither did age nor gender affect the survival time in pCCA (Figures 2A,B, Table 3).

**Table 3:** Univariate and multivariate analyses of survival in patients with perihilar cholangiocarcinoma.

**Feature**	**Univariate analysis**					**Multivariate analysis**		
	***n* = 488**	**OS (month)**	**5-year survival rates**	**HR (95% CI)**	***p*-value**	***n* = 331**	**HR (95% CI)**	***p-*value**
**Age (yr)**								
≤ 58	251	16	12%	1		-	-	-
>58	237	16	21%	0.84 (0.70–1.02)	0.068	-	-	-
**Gende**r								
Male	344	16	14.2%	1		-	-	
Female	144	15	14.6%	0.86 (0.69–1.06)	0.153	-	-	-
**Tumor size (range, 0.5–24 cm.)**								
Tumor *in situ*	25	119	80%	0.23 (0.13–0.44)	<0.001	-	-	-
≤ 4 cm	177	23	20.1%	1		177	1	
>4 cm	155	9	7.7%	1.87 (1.49–2.36)	<0.001	154	1.22 (0.94–1.61)	0.141
Unknown	131	-	-	-	-	-	-	-
**Surgical margin (R)**								
R0 (Tumor *in situ*)	25	119	80%	0.25 (0.14–0.47)	<0.001	-	-	-
R0	180	23	23.3%	1		119	1	
R1	283	9	6.3%	1.96 (1.60–2.40)	<0.001	212	1.22 (0.93–1.60)	0.156
**Growth pattern**								
ID (*in situ*)	25	119	80%	0.70 (0.34–1.47)	0.349	-	-	-
ID	39	88	76.9%	1		27	1	
PI	94	10	0%	14.67 (8.61–24.99)	<0.001	53	19.72 (9.20–42.24)	<0.001
MF	119	8	0%	22.66 (13.31–38.57)	<0.001	97	19.37 (9.02–41.60)	<0.001
ID+PI	54	35	27.8%	3.52 (2.03–6.08)	<0.001	30	4.67 (2.14–10.20)	<0.001
ID+MF	36	28	25%	4.72 (2.59–8.59)	<0.001	29	6.15 (2.77–13.68)	<0.001
PI+MF	82	10	0%	16.95 (9.87–29.11)	<0.001	63	16.01 (7.51–34.12)	<0.001
ID+PI+MF	39	28	17.9%	4.43 (2.50–7.83)	<0.001	32	4.94 (2.32–10.51)	<0.001
**Histological type**								
Tumor *in situ*	25	119	80%	0.21 (0.11-−0.39)	<0.001	-	-	-
Papillary carcinoma	196	19	17.3%	1		137	1	
Tubular carcinoma	180	16	12.8%	1.30 (1.05–1.61)	<0.05	139	0.86 (0.65–1.13)	0.276
Papillotubular carcinoma	19	12	15.8%	1.19 (0.73–1.97)	0.488	15	-	-
Adenocarcinoma, NOS	68	10	1.8%	2.38 (1.78–3.18)	<0.001	40	0.56 (0.25–1.27)	0.164
**Histological grade**								
Well-differentiated (*in situ*)	25	119	80%	0.19 (0.11–0.35)	<0.001	-	-	-
Well-differentiated	383	17	15.4%	1		280	1	
Moderately differentiated	56	11	1.7%	1.83 (1.37–2.44)	<0.001	32	2.31 (1.19–4.49)	<0.05
Poorly differentiated	24	5	0%	4.32 (2.81–6.65)	<0.001	19	4.99 (2.02–12.29)	<0.001
**T categories**								
T*is*	25	119	80%	0.31 (0.16–0.62)	<0.01	-	-	-
T1	40	30	27.5%	1		26	1	
T2a	125	24	17.6%	1.49 (1.00–2.22)	<0.05	82	0.83 (0.48–1.44)	0.515
T2b	197	15	13.2%	2.00 (1.36–2.94)	<0.001	153	0.97 (0.57–1.66)	0.920
T3	71	10	2.8%	3.54 (2.30–5.44)	<0.001	47	1.23 (0.68–2.20)	0.496
T4	30	6	0%	5.37 (3.21–8.99)	<0.001	23	1.38 (0.70–2.71)	0.348
**Lymph metastasis (N)**								
N0 (*in situ*)	25	119	80%	0.23 (0.12–0.42)	<0.001	-	-	-
N0	248	22	22.2%	1		168	1	
N1	182	12	3.3%	2.01 (1.63–2.47)	<0.001	135	1.37 (1.04–1.80)	<0.05
N2	33	7	0%	3.66 (2.50–5.37)	<0.001	28	2.18 (1.39–3.44)	<0.01
**Distal metastasis (M)**								
M0 (*in situ*)	25	119	80%	0.19 (0.10–0.35)	<0.001	-	-	-
M0	415	16	14.7%	1		294	1	
M1	48	6	0%	3.49 (2.55–4.79)	<0.001	37	2.11 (1.44–3.10)	<0.001

Non-invasive CCA served as a baseline for good survival time against which all other clinicopathological features were analyzed. Tumor size was available in 350 patients (Figure 2C, Table 3). Survival analysis showed that patients with tumor size >4 cm had significantly shorter survival time and 5-year survival rate than those with tumor size ≤ 4 cm (OS = 9 vs. 23 mo., 5y = 7.7% vs. 20.1%, *p* < 0.001), while those with tumor size ≤ 4 cm had significantly shorter survival time than those with tumor *in situ* (OS = 23 vs. 119 mo., 5y = 20.1 vs. 80%, *p* < 0.001).

Growth patterns identified by the surgeon-pathologist team included ID in non-invasive/*in situ* CCA, ID, PI, and MF, and combinations of ID+PI, ID+MF, PI+MF, and ID+PI+MF (Figure 2D, Table 3). Results showed that ID (OS = 88 mo.) had a significantly better survival time than PI (OS=88 vs 10 mo., 5y = 76.9 vs. 0%, *p* < 0.001), MF (OS = 88 vs. 8 mo., 5y = 76.9 vs. 0%, *p* < 0.001), ID+PI (OS = 88 vs. 35 mo., 5y = 76.9 vs. 27.8%, *p* < 0.001), ID+MF (OS = 88 vs. 28 mo., 5y = 76.9 vs. 25%, *p* < 0.001), PI+MF (OS = 88 vs. 10 mo., 5y = 76.9 vs. 0%, *p* < 0.001), and ID+PI+MF (OS = 88 vs. 28 mo., 5y = 76.9 vs. 17.9%, *p* < 0.001). However, there was no significant difference between ID and ID in non-invasive CCA (OS = 88 vs. 119 mo., 5y = 76.9 vs. 80%, *p* = 0.349).

Surgical margin (R) status was assessed microscopically (Figure 2E, Table 3). Patients having invasive CCA with positive surgical margins (R1) had survival time and rate significantly shorter than those with free surgical margins (R0) (OS = 9 vs. 23 mo., 5y = 6.3 vs. 23.3%, *p* < 0.001). In addition, the survival time of R0 in invasive CCA was significantly lower than R0 in non-invasive (tumor *in situ*) CCA (OS = 23 vs. 119 mo., 5y = 23.3 vs. 80%, *p* < 0.001).

Histological type was specified by the pathologist (Figure 2F, Table 3). Tubular and adenocarcinoma, NOS, types, had significantly shorter survival time and rate than papillary type, which was used as the reference group (OS = 16 vs. 19 mo., 5y = 12.8 vs. 17.3% *p* < 0.05) and (OS = 10 vs. 19 mo., 5y = 1.8 vs. 17.3%, *p* < 0.001), respectively. Additionally, the survival time of papillary type was shorter than histological types with *in situ* papillary or tubular types (OS = 19 vs. 119 mo., 5y = 17.3 vs. 80%, *p* < 0.001). No significance was recorded between papillary and papillotubular types (OS = 19 vs. 12 mo., 5y = 17.3 vs. 15.8%, *p* = 0.488).

Histological grades comprised well-, moderately, and poorly differentiated pCCA (Figure 2G, Table 3). The OS of patients with well-differentiated tumors was significantly higher than those with moderately (OS = 17 vs. 11 mo., 5y = 15.4 vs. 1.7%, *p* < 0.001) and poorly differentiated tumors (OS = 17 vs. 5 mo., 5y = 15.4 vs. 0%, *p* < 0.001). The OS of well-differentiated invasive CCA was markedly shorter than those with well-differentiated *in situ* tumors (OS = 17 vs. 119 mo., 5y = 15.4 vs. 80%, *p* < 0.001).

T categories according to the eighth AJCC staging system comprise T*is*, T1, T2a, T2b, T3, and T4 (Figure 2H, Table 3). Results of the survival analysis, using T1 as reference group, showed that T1 (OS = 30 mo.) was markedly better than T2a (OS = 30 vs. 24 mo., 5y = 27.5 vs. 17.6%, *p* < 0.05), T2b (OS = 30 vs. 15 mo., 5y = 27.5 vs. 13.2%, *p* < 0.001), T3 (OS = 30 vs. 10 mo., 27.5 vs. 2.8%, *p* < 0.001), and T4 (OS = 30 vs. 6 mo., 5y = 27.5 vs. 0%, *p* < 0.001), while OS of T1 was lower than T*is* (OS = 30 vs. 119 mo., 5y = 27.5 vs. 80%, *p* < 0.01).

As for lymph node metastasis (N) and distant metastasis (M) (Figures 2I,J, Table 3), results showed that patients who were either N1/N2 or M1 positive had remarkably shorter survival times than those with negative results (OS = 12 vs 22 mo., 5y = 3.3 vs. 22.2%, *p* < 0.001), (OS = 7 vs. 22 mo., 5y = 0 vs. 22.2%, *p* < 0.001), and (OS = 6 vs 16 mo., 5y = 0 vs. 14.7%, *p* < 0.001), respectively. As expected, invasive CCA with negative N and M status correlated with significantly shorter survival times than *in situ* tumors (OS = 22 vs. 119 mo., 5y = 22.2 vs. 80%, *p* < 0.001) and (OS = 16 vs. 119 mo., 5y = 14.7 vs. 80%, *p* < 0.001), respectively.

Significant factors such as age, tumor size, surgical margin, growth patterns, histological type, histological grade, and TNM categories were identified in the univariate analysis. These factors were selected for further investigation by multivariate analysis to identify independent factors for prognostication in pCCA. For multivariate analysis, only invasive pCCA was analyzed after removing non-invasive/*in situ* cases.

### Multivariate analysis of significant pathological features from univariate analysis

Univariate analysis of 331 patients identified tumor size, surgical margin, growth patterns, histological type, histological grade, T category, lymph node metastasis, and distant metastasis as having significantly affected survival times of patients with pCCA. Further analysis by multivariate analysis revealed that growth patterns, histological grade, lymph node metastasis, and distant metastasis were independent risk factors for the prediction of poor outcomes in patients with pCCA (Table 3).

For growth patterns, ID was used as a reference group for comparison with other growth patterns. Multivariate analysis showed that PI and MF had HR significantly higher than ID (HR = 19.72 and 19.37, *p* < 0.001), respectively. This finding was also found in the combination types; ID+PI, ID+MF, PI+MF, and ID+PI+MF showed HR significantly higher than ID (HR = 4.67, 6.15, 16.01, and 4.94, *p* < 0.001), respectively. We observed that OS of patients was increased when the growth patterns contained an ID component (ID+PI, ID+MF, and ID+PI+MF); henceforth, labeled as ID mixed types. Therefore, it was postulated that patients with pCCA with growth patterns lacking an ID component tended to do poorly. This finding suggested that the presence of ID components in growth patterns was a favorable prognostic factor for patients with pCCA. The corollary was that the lack of ID component was a poor prognostic factor.

For histological grades, the moderately and poorly differentiated tumors had HR markedly higher than the well-differentiated reference group (HR = 2.31, *p* < 0.05; and HR = 4.99, *p* < 0.001), respectively.

Lymph node metastasis and distant metastasis were consistent risk factors for poor prognosis. Multivariate results showed that positive lymph nodes (N1, N2) and distant metastasis had HR significantly greater than when they were negative (HR = 1.37 and 2.18, *p* < 0.05 and 0.01; and HR = 2.11, *p* < 0.001), respectively.

This study showed that growth patterns (PI, MF, and PI+MF), histological grade (moderate and poor differentiation), lymph node metastasis, and distant metastasis were prognostic risk factors for poor survival in patients with pCCA. Interestingly, growth patterns impacted the survival of patients with pCCA with high HR, and survival time changed between tumors with or without ID components. Therefore, this study aims to propose the incorporation of growth patterns for clustering patients with pCCA as an alternative tool for classifying such patients in our cohort. The limited number of cases in the higher histological grade groups precluded the incorporation of histological grade into the modification of the eighth AJCC staging system.

### Stratification performance of the eighth AJCC/UICC staging system

**Figure 3:**
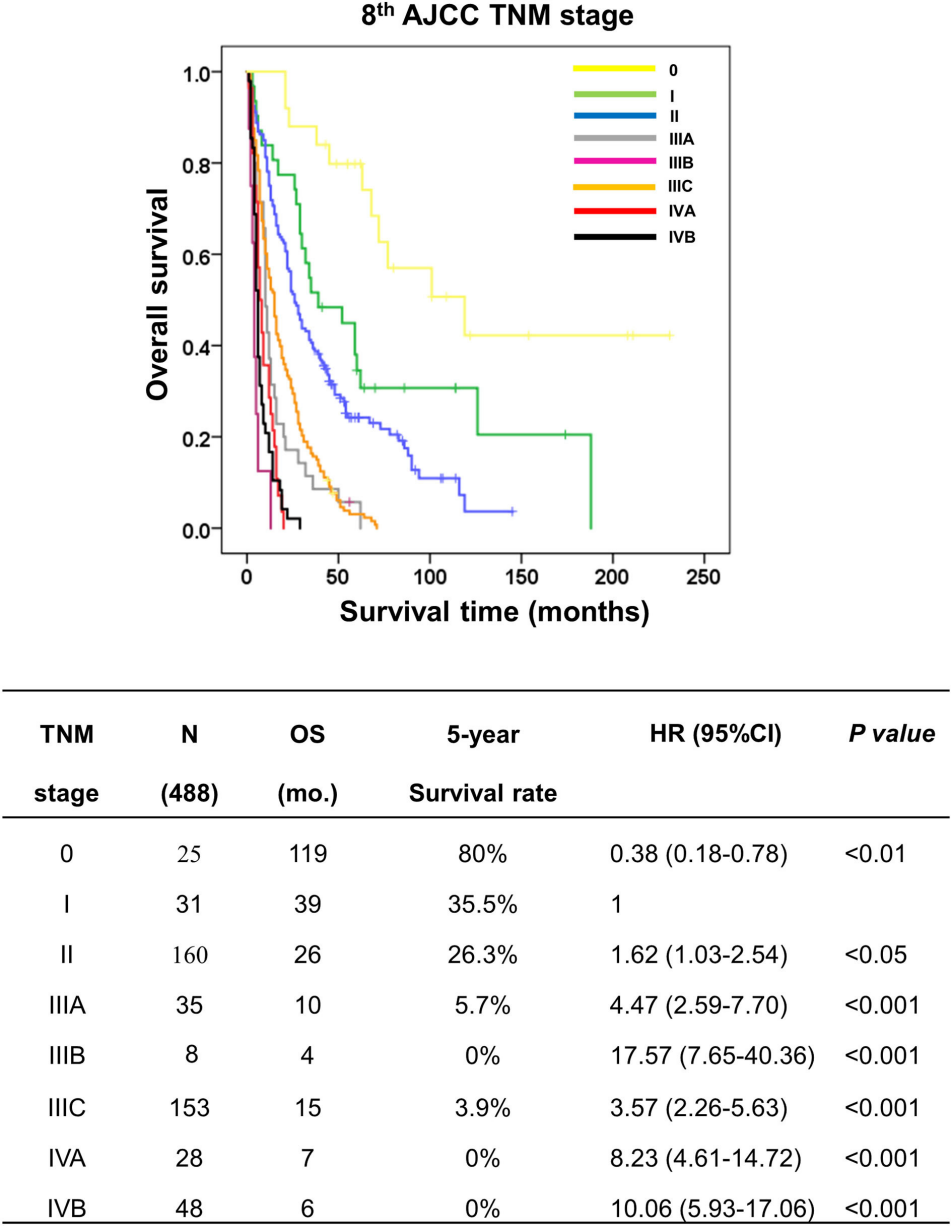
Kaplan–Meier curve represented the overall survival of patients with perihilar cholangiocarcinoma with TNM stages by the eighth AJCC staging system. The table represented the outcomes of patients with TNM stage by the eighth AJCC staging system, namely, TNM stage, number of cases, overall survival, 5-year survival rate, hazard ratio, and *p*-value.

To investigate the stratification performance of the eighth AJCC staging system, TNM staging was performed to classify pCCA according to the AJCC staging manual (Table 1). The results showed stage 0 (*n* = 25, OS = 119 mo., 5y = 80%), I (*n* = 31, OS = 39 mo., 5y = 35.5%), II (*n* = 160, OS = 26 mo., 5y = 26.3%), IIIA (*n* = 35, OS = 10 mo., 5y = 5.7%), IIIB (*n* = 8, OS = 4 mo., 5y = 0%), IIIC (*n* = 153, OS = 15 mo., 5y = 3.9%), IVA (*n* = 28, OS = 7 mo., 5y = 0%), and IVB (*n* = 48, OS = 6 mo., 5y = 0%). The stratification performance of the eighth AJCC staging system was inefficient in classifying and separating certain stages of patients with pCCA. The survival times and rates were equivocal and there was no significant difference between stages IIIA and IIIB; in fact, the survival times were shorter than for IIIC. Furthermore, stage IIIB had the shortest survival time when compared to IVA and IVB (Figure 3). This poor performance of the eighth AJCC staging system may impact prognosis and treatment decisions.

This study proposed an alternative staging system by employing prognostic factors from multivariate analysis to improve classification performance for pCCA. Our findings revealed that growth pattern has the highest impact on the survival of patients with pCCA. We hypothesize that the inclusion of a growth pattern subgroup may provide the necessary improvement required for pCCA classification.

### Subgroup analysis of growth pattern and lymph node status

**Figure 4:**
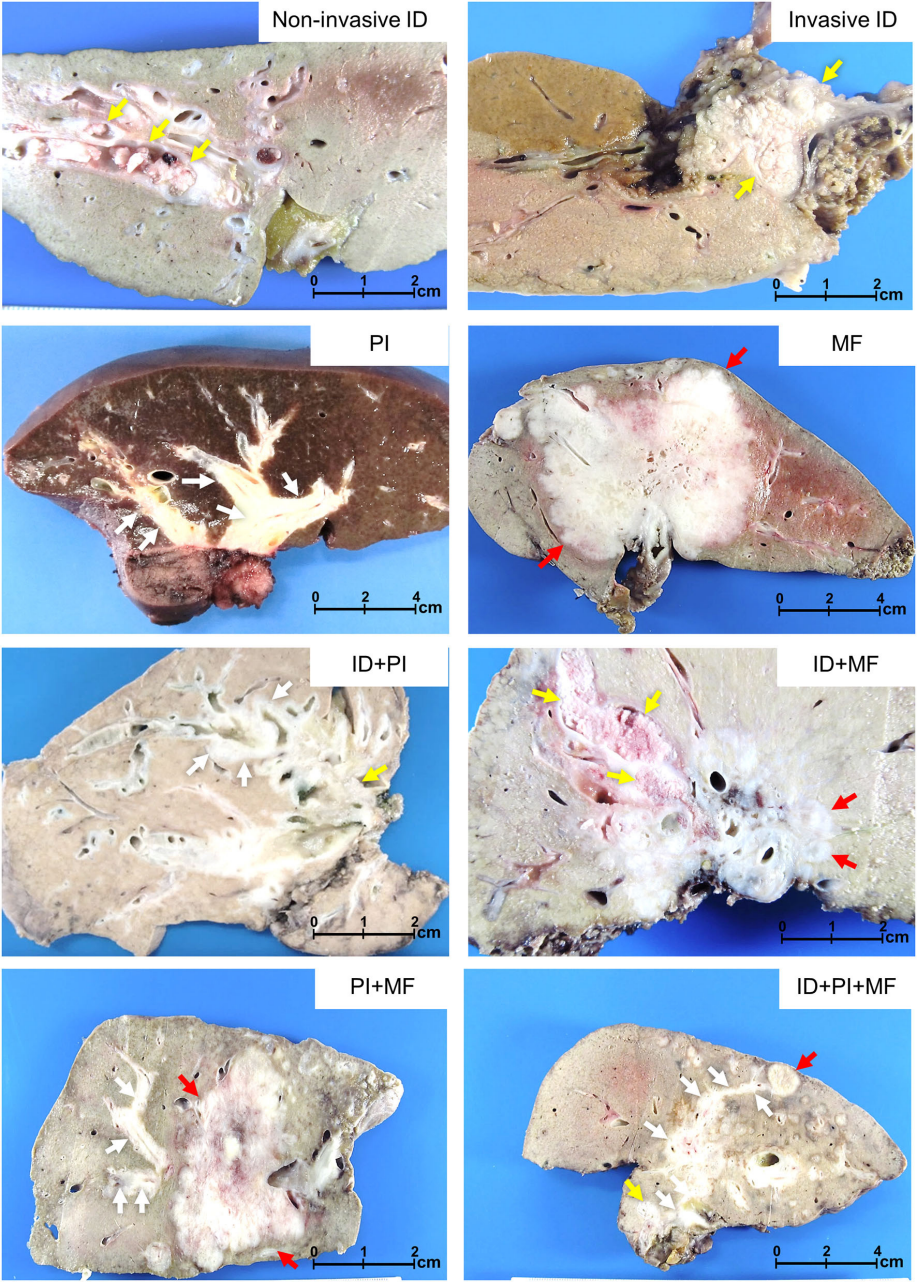
Growth patterns of perihilar cholangiocarcinoma. Non-invasive intraductal (ID), invasive ID, periductal infiltrating (PI), mass-forming (MF), and mixed types comprising ID+PI, ID+MF, PI+MF, and ID+PI+MF. Yellow, white, and red arrows indicate ID, PI, and MF, respectively.

There were seven growth patterns based on various morphological proportions, comprising ID, PI, MF, ID+PI, ID+MF, PI+MF, and ID+PI+MF (Figure 4), and they were associated with different survival outcomes. Based on survival outcomes, the patients were classified into three groups comprising well (ID, OS = 88 mo.), moderate (ID+PI, ID+MF, and ID+PI+MF, OS = 35, 28, and 28 mo., respectively), and poor (PI, MF, and PI+MF, OS = 10, 8, and 10 mo., respectively) outcomes (Figure 2D, Table 3). Notably, the survival outcomes of patients were favorably associated with the presence of the ID component. Therefore, on the basis of the ID component, we stratified growth patterns into three subgroups: ID, ID mixed types (ID+PI, ID+MF, and ID+PI+F), and without ID mixed types (PI, MF, and PI+MF). In addition, this investigation excluded N2M0 (*n* = 28) and M1 (*n* = 48) status because they represent late-stage disease and are well-known poor prognostic factors for pCCA.

This investigation aims to subclassify growth patterns with N0 and N1 status and to determine whether N status has any effect on influencing the survival time of various growth patterns when N status changes. To demonstrate this hypothesis, the growth pattern was divided into seven groups, comprising ID (*in situ*) as a baseline for good prognosis, ID/N0, ID/N1, ID mixed type/N0, ID mixed type/N1, without ID mixed type/N0, and without ID mixed type/N1.

The survival analysis showed that ID/N0 had OS and 5-year survival rate better than ID/N1 (OS = 116 vs. 51 mo., 5y = 83.3% vs. 50%, *p* < 0.01), ID mixed type/N0 (OS = 116 vs. 40 mo., 5y = 83.3 vs. 35.7%, *p* < 0.001), ID mixed type/N1 (OS = 116 vs 24 mo., 5y = 83.3 vs. 5.3%, *p* < 0.001), without ID mixed type/N0 (OS = 116 vs. 11 mo., 5y = 83.3 vs. 0%, *p* < 0.001), and without ID mixed type/N1 (OS = 116 vs. 40 mo., 5y = 83.3 vs. 0%, *p* < 0.001), while no significant difference was observed when compared with ID (*in situ*) (OS = 116 vs. 119 mo., 5y = 83.3 vs. 80%, *p* = 0.888).

**Figure 5:**
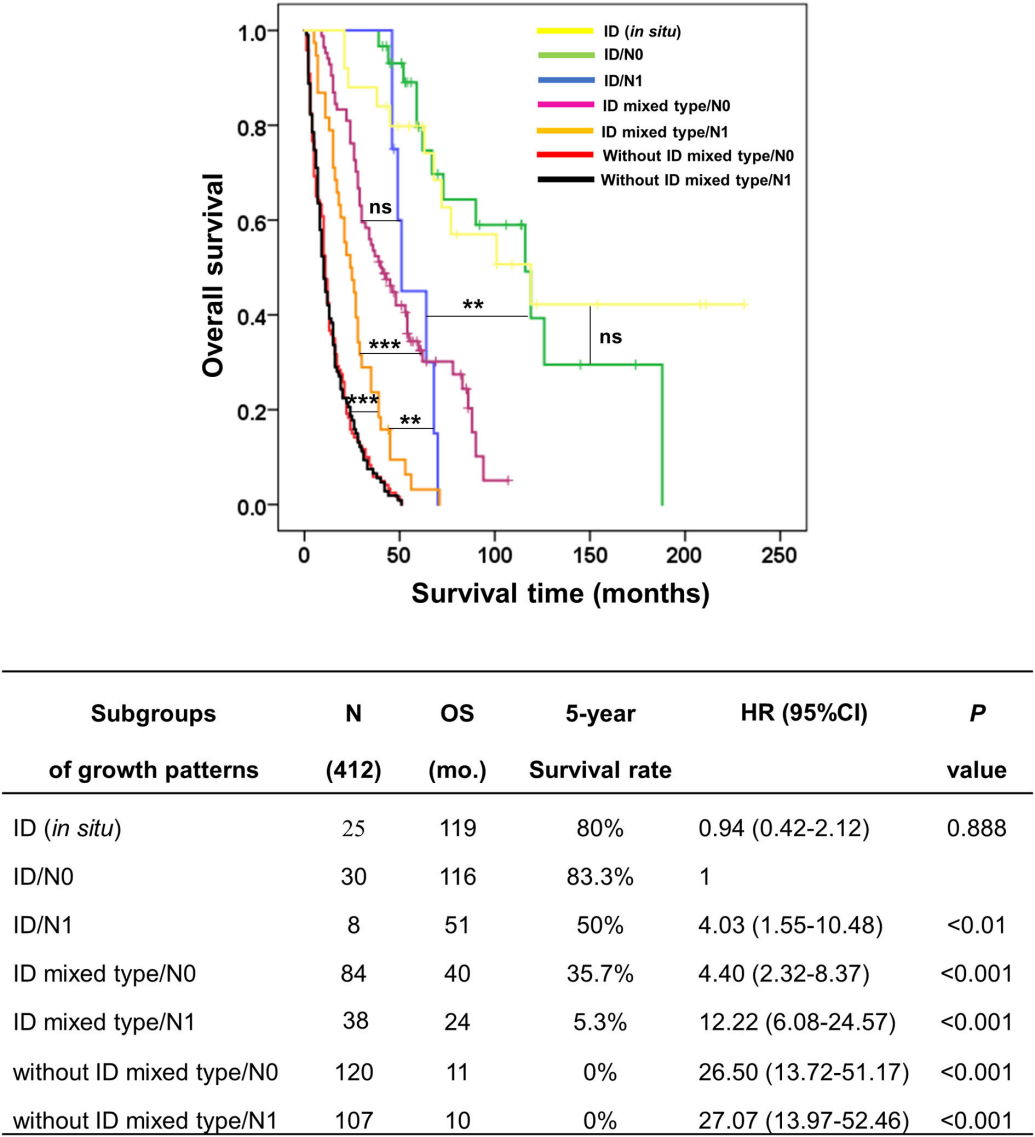
Kaplan–Meier curve represented the overall survival of subgroup coordinating growth pattern and lymph node status (N0 and N1) in patients with perihilar cholangiocarcinoma. The table represented the outcomes of subgroup analysis, namely, subgroups of growth patterns, number of cases, overall survival, 5-year survival rate, hazard ratio, and *p*-value. **p < 0.01, ***p < 0.001, and ns = no statistical significance.

Additionally, the comparison between subgroups found that ID/N1 and ID mixed type/N0 were not significantly different, while both ID/N1 and ID mixed type/N0 were markedly different when compared with ID mixed type/N1 (*p* < 0.01 and 0.001, respectively). Furthermore, ID mixed type/N1 had obviously better survival time than both without ID mixed type/N0 (*p* < 0.001) and without ID mixed type/N1 (*p* < 0.001), while the comparison of ID mixed type/N0 and without ID mixed type/N1 showed no significant difference (Figure 5). This information supported the hypothesis that the survival time of growth pattern-classified patients was decreased when N1 appeared.

### Creating growth pattern (G) category of the KKU staging system

From Figure 5, we found that N1 could upgrade the aggressive behavior of growth pattern-classified pCCA, such as in ID (N0/N1) and ID mixed type (N0/N1), while no difference was found in without ID mixed type (N0/N1). Therefore, we generated a new G category to replace the T category of the eighth AJCC staging system. The G category was created according to the survival analysis of subgroups of growth patterns without lymph node positivity.

**Figure 6:**
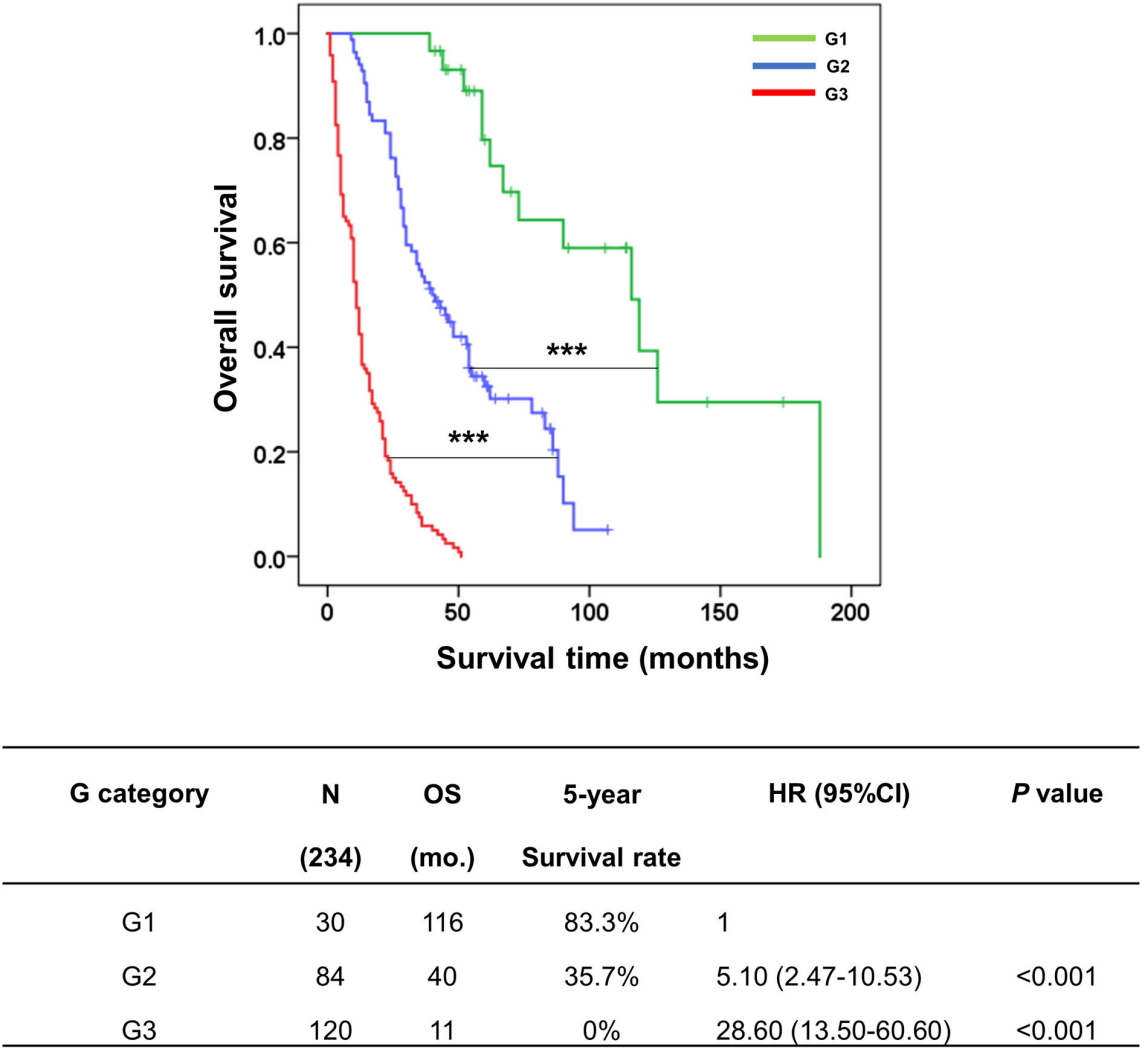
Kaplan–Meier curve represented the overall survival of the G category in the KKU staging system for classifying patients with perihilar cholangiocarcinoma. The table represented the outcomes of patients with G category classification, namely, groups, number of cases, overall survival, 5-year survival rate, hazard ratio, and *p*-value. ***p < 0.001.

There were three classes of the G category in this study, comprising G1 (ID/N0, *n* = 30), G2 (ID mixed type/N0, *n* = 84), and G3 (without ID mixed type/N0, *n* = 120). The survival analysis showed that G1 had OS and survival rate markedly better than G2 (OS = 116 vs. 40 mo., 5y = 83.3 vs. 35.7%, *p* < 0.001) and G3 (OS = 116 vs. 11 mo., 5y = 83.3 vs. 0%, *p* < 0.001). Moreover, the comparison of survival time for each G category revealed a clear separation between each stage; G2 vs G3 (*p* < 0.001) (Figure 6).

### Classification of patients with perihilar cholangiocarcinoma using the KKU staging system

According to Figures 5, 6, we proposed the KKU staging manual for classifying patients with pCCA (Table 4). G category was incorporated together with N and M categories (according to the eighth AJCC staging system) into the KKU stage: I (G1/N0/M0), II (G1/N1/M0 or G2/N0/M0), IIIA (G2/N1/M0), IIIB (G3/M0/M0 or G3/N1/M0), IVA (anyG/N2/M0), and IVB (anyG/anyN/M1). The results showed that patients with pCCA can be classified into seven stages, comprising KKU stage 0 (*n* = 25), I (*n* = 30), II (*n* = 92), IIIA (*n* = 38), IIIB (*n* = 227), IVA (*n* = 28), and IVB (*n* = 48). KKU stage 0 represents non-invasive tumor (carcinoma *in situ*//high-grade dysplasia) with good survival, while KKU stage I is a baseline for a good prognosis of invasive tumor which had similar outcomes as stage 0 (OS = 116 vs. 119 mo., 5y = 83.3 vs. 80%, *p* = 0.888).

**Table 4:** Definition of GNM categories of the KKU staging manual.

**G category (G)**	Lymph node metastasis (N)^*^	Distant metastasis (M)^*^
T*is* = Carcinoma in *situ*//high-grade dysplasia^*^	N0 = Negative lymph node	M0 = No distant metastasis
G1 = Pure intraductal growth pattern (ID)	N1 = One to three positive lymph nodes	M1 = Distant metastasis
G2 = With ID mixed types (ID+PI, ID+MF, ID+PI+MF)	N2 = Four or more positive lymph nodes	
G3 = Without ID mixed types (PI, MF, PI+MF)		
**KKU staging system (GNM stage)**		
Stage I	G1/N0/M0	
Stage II	G1/N1/M0, G2/N0/M0	
Stage IIIA	G2/N1/M0	
Stage IIIB	G3/N0/M0, G3/N1/M0	
Stage IVA	AnyG/N2/M0	
Stage IVB	AnyG/anyN/M1	

* *According to the eighth AJCC staging system*.

*G1 was the growth pattern; ID*.

*G2 was the growth pattern; ID+PI, ID+MF, and ID+PI+MF*.

*G3 was the growth pattern; PI, MF, and PI+MF*.

The stratification performance demonstrated good separation between stages with overall survival and survival rate of KKU stage I being significantly better than for II (OS = 116 vs. 46 mo., 5y = 83.3 vs. 37%, *p* < 0.001), IIIA (OS = 116 vs. 24 mo., 5y = 83.3 vs. 5.3%, *p* < 0.001), IIIB (OS = 116 vs. 11 mo., 5y = 83.3 vs. 0%, *p* < 0.001), IVA (OS = 116 vs. 7 mo., 5y = 83.3 vs. 0%, *p* < 0.001), and IVB (OS = 116 vs. 6 mo., 5y = 83.3 vs 0%, *p* < 0.001).

**Figure 7:**
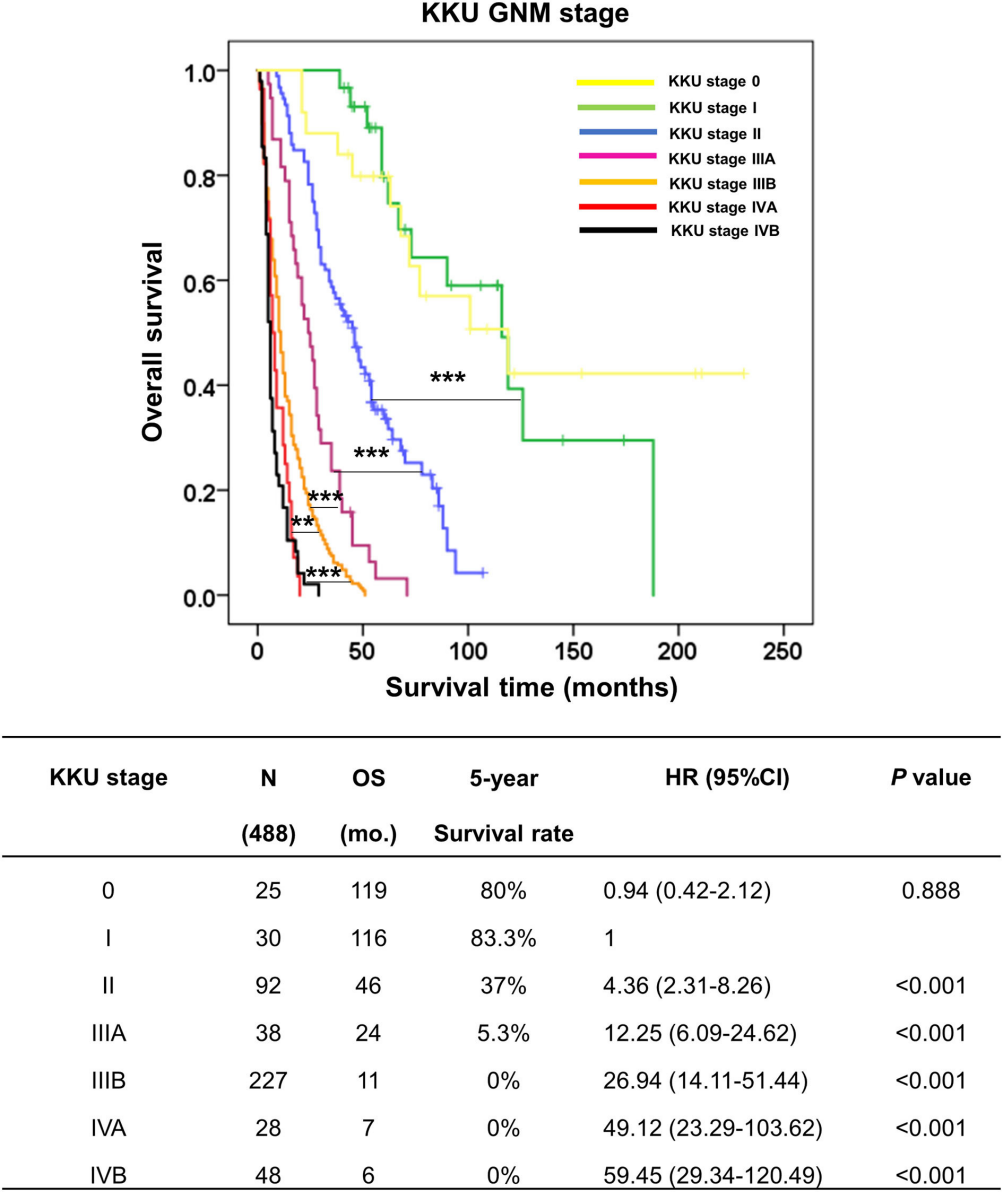
Kaplan–Meier curve represented the overall survival of perihilar cholangiocarcinoma patients with GNM stages by the KKU staging system. The table represented the outcomes of patients with GNM stage by the KKU staging system, namely, KKU stage, number of cases, overall survival, 5-year survival rate, hazard ratio, and p-value. **p < 0.01 and ***p < 0.001.

Moreover, the comparison of each stage showed good separation: II vs IIIA (*p* < 0.001), IIIA vs IIIB (*p* < 0.001), and IIIB vs. IVA (*p* < 0.01), and IVB (*p* < 0.001) (Figure 7). These findings support the contention that the KKU staging system has high performance to classify patients with pCCA in Thailand; thus, providing an alternative tool to overcome the weak classification ability of the eighth AJCC staging system.

## Discussion

Nowadays, curative therapy for pCCA is limited to the surgical strategy with poor 5-year survival rates (11–13). Our study showed the survival rates of patients with pCCA at 1, 3, and 5 years after partial hepatectomy to be 58, 27, and 11.5%, respectively (Supplementary Figure 1). Previous reports have suggested that the major issue was late diagnosis with advanced disease (10, 11). About 56% of our cohort of patients with pCCA between 2002 and 2017 presented with advanced disease–40% stage III and 16% stage IV, based on the eighth AJCC staging system. This translated into a higher rate of surgical margin involvement with R1 as high as 58% when compared to the reported range of 11–39% in other cohorts (26, 27, 29, 31, 32, 36, 37). This clearly shows that early diagnosis is crucial for accurate clustering and treatment.

The current stratification tool for clustering pCCA is the eighth edition of the AJCC staging system (21). Several reports have suggested that the updated edition provided a better classification and stratification performance than the seventh edition (22–25, 38). Ruzzenente et al. (22) compared both editions of the AJCC staging system in 214 patients who underwent surgery for pCCA at two Italian tertiary referral hepatobiliary centers. Based on the seventh edition, the 5-year OS rates were stage I (71%), II (34%), and IV (34%), while there were no patients who survived 5 years in stage IIIA, IIIB, and IV. The eighth edition, however, appeared to have improved discriminatory ability with consistent classification for 5-year OS in stage I (71%), II (35%), and better discrimination for stage IIIA (23%), IIIB (19%), and IIIC (22%). Interestingly, there was no 5-year OS for stages IVA and IVB. The C-index representing discriminatory performance was higher for the eighth than the seventh edition (0.624 vs. 0.619). Similarly, Lee et al. (24) showed in 348 patients who underwent hepatectomy that the stratification by the eighth edition of each group of T classification improved the clustering of patients with T3 and T4 when compared to the seventh edition. The eighth AJCC classification showed increase in OS of T3 (T1, 42.6%; T2, 31.2%; T3, 13.9%; T4, 15.1%; T1 vs. T2, *p* = 0.260; T2 vs. T3, *p* = 0.001; T3 vs. T4, *p* = 0.996), and decrease of OS of T4 and when each T stage was separated, especially T3 and T4. These results were greater than by classification by the seventh AJCC staging system (T1, 41.0%; T2, 30.5%; T3, 9.1%; T4, 25.7%; T2 vs. T3, *p* < 0.001; T3 vs. T4, *p* = 0.013). Gaspersz et al. (23) analyzed the eighth AJCC staging system in 248 patients with pCCA by separating them into (i) patients with curative-intent resection (18.1%) and (ii) patients with non-resection due to metastasis (81.9%). The prognostic accuracy (C-index) was performed for the comparison of stratification performance. The results showed that the eighth edition had C-index higher than seventh edition for both curative-intent resection (0.67 vs. 0.65) and non-resection (0.58 vs. 0.57).

Although the prognostic predictability of the eighth edition is improved, the overall performance remains unsatisfactory (22–25). Accordingly, when this study applied the eighth AJCC staging system to classify 488 patients who had undergone partial hepatectomy for pCCA, the OS and 5-year survival rate results were not consistent and were ambiguous for each stage. Furthermore, the survival analysis displayed ambiguous stratification in stages III and IV (OS = IIIC>IIIA>IVA>IVB>IIIB)—IIIB had poor survival time less than IIIA, IIIC, IVA and IVB; and IIIA was less than IIIC (Figure 3). These results may lead to errors in diagnosis, prognosis, and treatment plans.

Numerous studies have suggested additional factors to modify the eighth AJCC staging system for suitable and precise prognostic predictability of pCCA (26–32). Prognostic factors which are independent factors from the multivariate analysis are favored, one of which is invasive tumor thickness (ITT). The new T classification based on ITT better separates each T stage than the eighth AJCC staging system (26). Oba et al. (27) confirmed that although they used different ITT cut-off values, T classification by ITT was able to distinguish the T stage better than the eighth AJCC staging system. Zhang et al. (28) proposed tumor size as a prognostic factor. They suggested that larger tumor size (range, 22–33 mm or ≥33 mm) is associated with poor outcomes, such as advanced T stage, more positive regional lymph nodes, and more frequent vascular invasion. For this study, we applied the growth pattern to improve the predictive accuracy of the prognosis of the eighth AJCC staging system (18, 39). Moreover, Cheng et al. (40) proposed the incorporation of serum tumor markers; carcinoembryonic antigen (CEA) and carbohydrate antigen 19-9 (CA 19-9), into the TNM staging system to form the mTNM staging system. The classification performance of mTNM showed a better ability to separate each stage than the TNM staging system.

In this study, growth patterns, histological grades (moderate and poor differentiation), lymph node, and distant metastasis were found to be independent factors for predicting poor survival of patients with pCCA. Interestingly, the growth patterns that included PI, MF, ID+PI, ID+MF, PI+MF, and ID+PI+MF had an HR markedly higher than the ID growth pattern, which is viewed as a favorable prognostic marker in patients with iCCA (15, 18, 41). In this cohort, almost all the pCCA cases were found in the intrahepatic location. We demonstrated a spectrum of growth patterns—ID, PI, and MF, and combined types comprising ID+PI, ID+MF, PI+MF, and ID+PI+MF. The proportion of combined types was considered by a surgeon-pathologist team with a 20% cut-off. Based on multivariate analysis, growth pattern was considered a major prognostic factor for poor survival of patients with pCCA (Table 3). Moreover, although histological grade (moderate and poor differentiation) was an independent factor in the multivariate analysis, the data were not robust enough to support inclusion in the new staging system.

**Figure 8:**
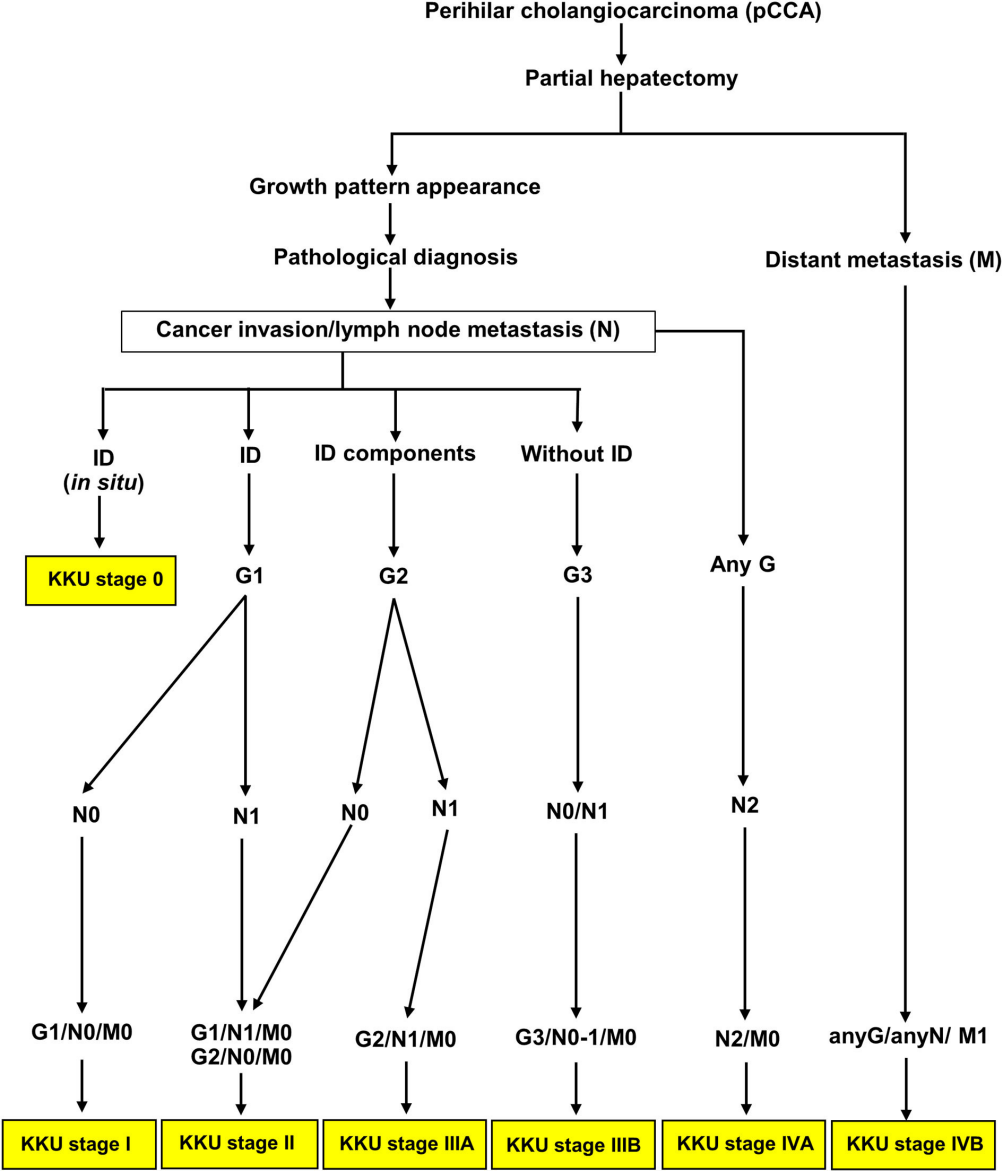
Schematic of the KKU staging system for classifying patients with perihilar cholangiocarcinoma.

In applying prognostic factors to generate a new KKU staging system, we created a new G category to replace the T category of the eighth AJCC tagging system. The patients with pCCA were clustered into three groups comprising G1 (ID), G2 (ID mixed types: ID+PI, IP+MF, ID+PI+F), and G2 (PI, MF, without ID mixed type—PI+MF). The G category showed good separation—G1 vs. G2 and G2 vs. G3; and OS and survival rate were decreased when G was increased (OS and 5-year survival rate = G1>G2>G3). The KKU staging system retained the N and M categories of the eighth AJCC staging system because both categories were independent factors in this study. Moreover, we found that all growth patterns showed shorter survival when N1, N2, and M1 status appeared compared to those with negative status (Figure 5); except for N1 without ID mixed type (PI, MF, and PI+MF) which showed no difference when N0 vs. N1 was compared. G3 showed poor outcomes irrespective of N0 or N1. Supplementary Table 1 showed that G3 was significantly correlated with tumor size, high histological grade, and high T category in the eighth AJCC staging system; hence, the poor survival. Previous reports support our findings that PI and MF were correlated with poor survival of patients with CCA, while ID favored a good prognosis (15, 18, 42, 43). Therefore, the GNM stage by the KKU staging manual was applied to classify patients with pCCA (Table 4), with the resultant seven stages: KKU stage 0, I, II, IIIA, IIIB, IVA, and IVB. The performance of the KKU staging system (Figure 7) was superior to the eighth AJCC staging system (Figure 3) for patients with pCCA in Thailand. We hope this improvement will provide for a more precise prognosis, diagnosis, and choice of treatment plan. Therefore, we proposed the KKU staging system as an alternative tool for classifying patients with pCCA (Figure 8).

Our study has three major strengths. Northeast Thailand has the highest incidence of CCA worldwide. Furthermore, pCCA constituted the majority of the patients with CCA; thus, giving us a sizeable cohort for statistically significant analysis. Routine recording of growth pattern data has also enabled us to analyze and conclude that PI and MF patterns forebode poor prognostic outcomes (1, 15, 18).

However, there are several limitations to this study. The proposed KKU staging system was applied in a single institution in Northeast Thailand. Overall survival and not disease-specific survival were studied. The small number of patients in certain stages, such as stage IIIB, does not adequately represent the rest of the patient population. Histological grade and serum tumor markers (CEA and CA 19-9) are commonly reported characteristics in cancer prognostication and are important independent risk factors for stratifying patients; further analysis with a larger number of cases would be performed for subsequent incorporation into the KKU model. Post-operative complications are important and highly impact prognosis; however, this information was not available in our database. There was also no available follow-up data on adjuvant therapy, thereby significantly curtailing the evaluation of surgical margin status. Finally, the internal and external validation of the KKU staging system is needed to confirm and verify the discriminatory performance for the prognostic stratification of patients with pCCA.

For future perspective of this study, we believe that with validation from internal and external cohorts, the KKU staging system can be put into practice at our institute. Since growth pattern is routinely recorded in the pathological gross examination of CCA resection specimens, it may prove to be beneficial for stratification, prognostication, and planning for treatment or palliative care. Moreover, there is a prospect of applying the KKU staging system in a pre-operative setting. There are several studies in our institute, suggesting that pre-operative detection of ID lesions by radiology can be performed for early stages and for pre-invasive neoplasms of the bile ducts (44, 45). In 2017, Luvira V. and Eurboonyanun K. (co-authors in our study) investigated intraductal papillary neoplasms of the bile duct (IPNB) with survival correlation of patients with CCA via radiological–pathological staging. They created a new morphological classification of IPNB based on radiological–pathological appearance correlated with clinical findings, including outcomes. Their study showed that IPNB correlated with early stage and predicted survival very well (46, 47). In future, this study will be modified for incorporation into the KKU staging system in guiding the planning of surgical procedures.

In summary, in this study spanning 2002–2017, we updated significant information on the staging of pCCA. Approximately 56% of pCCA from Northeast Thailand presented with late-stage disease (TNM stage III, 40%) and distant metastasis (stage IV, 16%). All of these patients had poor survival outcomes. This study demonstrated that growth patterns, histological grade, lymph node, and distant metastasis are prognostic factors for poor outcomes. We propose a new KKU staging system by incorporating the growth pattern to modify and refine the eighth AJCC staging system for the evaluation of patients with pCCA. Our staging system showed prognostic ability better than the eighth AJCC staging system for the stratification of pCCA in Thailand. However, to put things in proper perspective, the KKU staging system is essentially employed for post-operative prognostic prediction and management planning; clinical pre-operative staging with radiological T of the eighth AJCC staging system still plays an important role in the pre-operative management plan. There is currently a prospective study where the KKU staging system is applied to patients with pCCA to validate its utility as a prognostic predictive tool and guidance to treatment options.

## Data availability statement

The datasets presented in this study can be found in online repositories. The names of the repository/repositories and accession number(s) can be found in the article/Supplementary material.

## Ethics statement

The studies involving human participants were reviewed and approved by the guidelines of the Declaration of Helsinki. This study was approved by the Ethics Committee for Human Research, Khon Kaen University (HE641499). The patients/participants provided their written informed consent to participate in this study.

## Author contributions

Conceptualization: PS-N, SK, CA, SS, and PI. Funding acquisition and supervision: SK. Sample collection and diagnosis: PS-N, SK, CA, SS, PI, WK, NK, AT, AJ, VT, TS, VL, KE, WL, and JP. Analysis and interpretation of data: PS-N, SK, CA, and PP. Writing—original draft: PS-N, SK, CA, SS, PI, and PP. Writing—review and editing: PS-N, SK, CA, SS, PI, WK, MT, AW, and PP. All authors approved the final version of the manuscript.

## Funding

We would like to acknowledge the Cholangiocarcinoma Screening and Care Program (CASCAP) under Cholangiocarcinoma Research Institute (CARI), Khon Kaen University, Khon Kaen, Thailand, and the National Research Council of Thailand through Fluke Free Thailand project, Khon Kaen University through Cholangiocarcinoma Research Institute to support funding.

## Conflict of interest

The authors declare that the research was conducted in the absence of any commercial or financial relationships that could be construed as a potential conflict of interest.

## Publisher's note

All claims expressed in this article are solely those of the authors and do not necessarily represent those of their affiliated organizations, or those of the publisher, the editors and the reviewers. Any product that may be evaluated in this article, or claim that may be made by its manufacturer, is not guaranteed or endorsed by the publisher.
