# Supplementary material for: Modification of the eighth AJCC/UICC staging system for perihilar cholangiocarcinoma: An alternative pathological staging system from cholangiocarcinoma-prevalent Northeast Thailand
Source: Front Med (Lausanne). 2022 Sep 30;9:893252. doi: 10.3389/fmed.2022.893252 (PMC9561347; doi:10.3389/fmed.2022.893252)
Supplement: Supplementary file 1 [file Data_Sheet_1.pdf]

frontiers

Supplementary Material

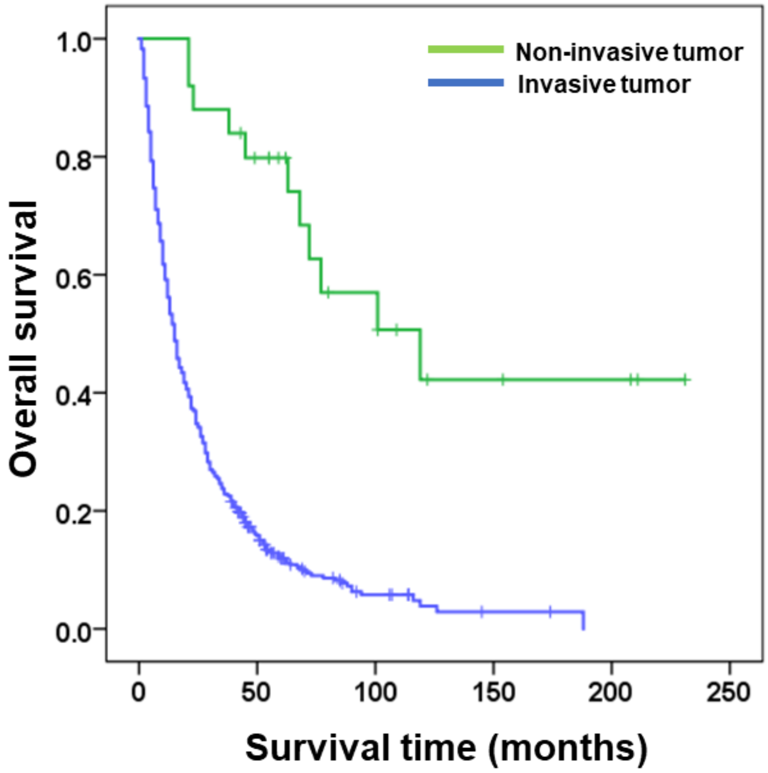

Supplementary Figure. Overall survival and 5-year survival rate of non-invasive and invasive tumors in perihilar cholangiocarcinoma patients

Supplementary Table. The correlation of G category of KKU staging system with tumor size, histology grade and T category of 8th AJCC staging system

**Feature**	**N**	**G category**			***P* value**
		**G1**	**G2**	**G3**	
Tumor size	159				<0.01
≤4 cm.	92	16	39	37	
>4 cm.	67	4	18	45	
Histological grade	234				<0.001
Well	187	30	79	78	
Moderately	31	0	5	26	
Poorly	16	0	0	16	
T category of 8th AJCC staging system	147				<0.001
T1	30	12	10	8	
T2	74	8	31	35	
T3	35	0	4	31	
T4	8	0	0	8	
